# Endoscopic lumbar discectomy: Experience of first 100 cases

**DOI:** 10.4103/0019-5413.62051

**Published:** 2010

**Authors:** Amit Jhala, Manish Mistry

**Affiliations:** Department of Spine Surgery, Chirayu Hospital, Ahmedabad, India

**Keywords:** Lumbar discectomy, microendo system, endoscopic lumbar discectomy

## Abstract

**Background:**

Various modalities of treatment from standard discectomy, microdiscectomy, percutaneous discectomy, and transforaminal endoscopic discectomy have been in use for lumbar intervertebral disc prolapse. The access to spine is kept to a minimum without stripping paraspinal muscles minimizing muscle damage by posterior interlaminar endoscopic approach. The aim of this study was to evaluate technical problems, complications, and overall initial results of microendoscopic discectomy.

**Materials and Methods:**

First 100 consecutive cases aged 19-65 years operated by microendoscopic dissectomy between August 2002 – December 2005 are reported. All patients with single nerve root lesions including sequestrated or migrated and selected central disc at L4-5 and L5-S1 were included. The patients with bilateral radiculopathy were excluded. All patients had preoperative MRI and first 11 patients had postoperative MRI to check the adequacy of decompression. Diagnostic selective nerve root blocks were done in selective cases to isolate the single root lesion when MRI was inconclusive (n=7). All patients were operated by a single surgeon with the Metrx system (Medtronics). 97 were operated by 18-mm ports, and only three patients were operated by 16-mm ports. Postoperatively, all patients were mobilized as soon as the pain subsided and discharged within 24–48 h postsurgery. Patients were evaluated for technical problems, complications, and overall results by modified Macnab criteria. Patients were followed up at 2, 6, and 12 weeks.

**Results:**

The mean follow up was 12 months (range 3 months – 4 years). Open conversion was required in one patient with suspected root damage. Peroperatively single facet removal was done in 5 initial cases. Minor dural punctures occurred in seven cases and root damage in one case. The average surgical time was 70 min (range 25-210 min). Average blood loss was 20-30 ml. Technical difficulties encountered in initial 25 cases were insertion of guide pin, image orientation, peroperative dissection and bleeding problems, and reaching wrong levels suggestive of a definitive learning curve. Postoperative MRI (n=11) showed complete decompression. Overall 91% of patients had good-to-excellent results, with four patients having recurrence of whom three were reoperated. Four patients had postoperative discitis. One of the patients required fusion for discitis and rest were managed conservatively. One patient had root damage to L5 root that had paresthesia in L5 region even on 4 years of follow-up.

**Conclusion:**

Microendoscopic discectomy is minimally invasive procedure for discectomy with early encouraging results. Once definite learning curve was over and expertise is acquired, the results of this procedure are acceptable safe and effective.

## INTRODUCTION

First discectomy was done by Oppenheim and Fedre Krause in 1906 though the first publication was done by Mixter and Bar.^1^,^2^ Since then laminectomy, hemilaminectomy and fenestration were introduced and are still being widely practiced world over. The lateral approach was evolved in 1964 with the introduction of intradiscal chymopapain injections.^3^ This was followed by the introduction of manual percutaneous discectomy by Hijikata^4^ followed by the introduction of automated percutaneous discectomy by Onik,^5^ laser nucleolysis^6^ and transdiscoscopy discectomy.^7^ The indications for these procedures have generally been limited to contained lumbar disc herniations, because lumbar radiculopathies secondary to large, free-fragment (noncontained) disc pathology leading to any kind of bony compression of the nerve root are still specific contraindications to percutaneous lumbar discectomy. Nevertheless, the idea of a percutaneous, even less invasive approach to a lumbar disc disease remained appealing.

Yasargil^8^ and Casper^9^ and Williams^10^ started the use of microscopes for posterior discectomy which limited the skin incision and less muscle and epidural scarring. Patients had less postoperative pain, early rehabilitation, and early return to work. Any disc pathology along with elements of bony lateral stenosis can be dealt with this approach. Ever since then, microdiscectomy has become a gold standard procedure. The advances in optics and instrument design have led to the successful application of less invasive surgical principles to the abdomen, the thoracic cavity, and several joints (knee, shoulder, and wrist), where the surgical efficacy is at least similar to that of the conventional, more invasive approaches, but with decreased hospital stays and shorter recovery times. The use of an endoscope for disc excision through posterior approach was introduced. The Microendo system allows the use of micro-instruments through a tube, making it possible, under endoscopic control, to perform a true discectomy. The incision size is further reduced with no paraspinal muscle cutting or detachment from their insertion but the muscles are dilated using their elasticity. This has further reduced the invasion to the paraspinal muscle and muscle scarring. This procedure is known as microendoscopic discectomy (MED). The new systems for endoscopic posterior discectomy are either a conic “freehand” working channel (the Endospine by J. Destandeau) or a tubular retractor (Metrx system, Medtronics), introduced by Foley and Smith.^11^

The author has been using the Metrx system on a regular basis since August 2002. This article retrospectively reviews the experience of first 100 cases from August 2002 till December 2005 for technical problems, complications and overall results.

## MATERIALS AND METHODS

A total of 100 consecutive cases aged 19-65 years operated by the MED procedure for L4-5 or L5-S1 PIVD from August 2002 to December 2005 were retrospectively evaluated for the result. All the cases were operated on by a single surgeon. The inclusion criteria were patients having lumbar disc prolapse with unilateral radiculopathy, on clinical evaluation, positive straight leg raising test and identification of a single nerve root lesion. Any patients with bilateral symptoms, double root involvement and cauda equina syndrome were excluded. On imaging, types of disc operated were all posterolateral discs including sequestrated (n=18) or migrated and selected central discs (n=8) with unilateral symptoms. All patients had preoperative MRI and first 11 patients had postoperative MRI to check the adequacy of decompression. Diagnostic selective nerve root blocks were done in selective cases (n=7) to isolate the single root lesion when MRI was inconclusive. All patients were operated only after proper conservative management for minimum 6 weeks which consisted of rest, modification of activities, physiotherapy and analgesics and anti-inflammatory drugs. The duration of symptoms ranged from 6 weeks to 8 years. The surgery was done by the Metrx system of Medtronic.

### Operative technique

All the procedures were done under general anesthesia. The patient was placed in prone position on either bolsters or a spinal frame, with the abdomen free and the spine flexed to open the interlaminar space. The surgeon stood on the side of the disc prolapse, the TV monitor was at the head end and IITV on the opposite side. A flexible arm assembly was attached to the operating table rail to hold the tubular retractor with an endoscope in a stable position, freeing the surgeon's hands.

**Figure 1:**
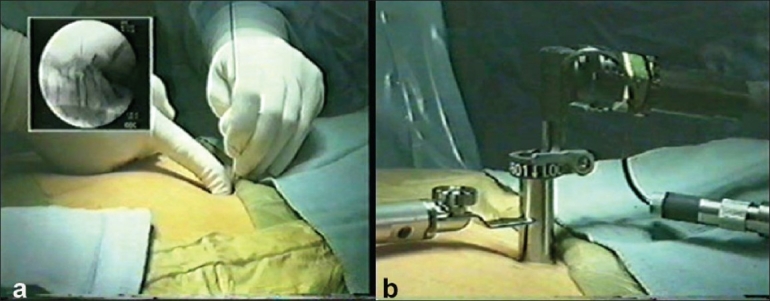
Intraoperative photograph shows (a) guide wire insertion (b) insertion of the tubular retractor and endoscope

The incision was marked in AP and lateral projection in IITV. The guide wire entry point is the key for the port and we checked the wire in IITV. In AP projection it should be at the inferior edge of the superior lamina and in lateral projection, it should be parallel to the disc space [Figure 1a]. Once the entry point was marked about 1-1.5 cm lateral to the midline, an 18-mm skin incision was made. The subcutaneous tissue and fascia were incised. The first dilator was introduced over the guide wire and the guide wire was then removed. The dilator was docked over the lamina and medial, lateral, superior, and inferior edges of the lamina were felt and the muscles were separated subperiosteally. The other dilators were sequentially introduced over the first dilator. This sequentially dilated the paraspinal muscles. The 18-mm tubular retractor was introduced over the last dilator and the final position was checked under IITV [Figure 1b].

The endoscope was connected to the coupler, camera, and light source. The whole assembly was introduced through the tubular retractor and the coupler was fixed to the outer margin of the tubular retractor.

**Figure 2:**
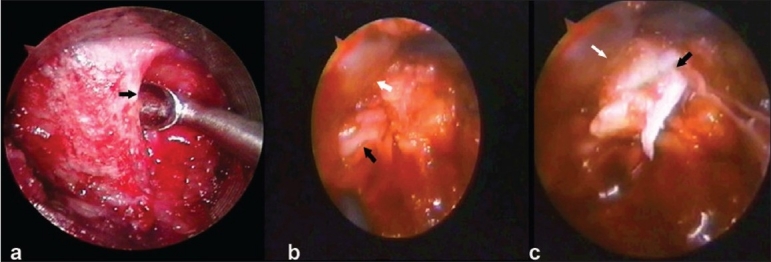
Intraoperative photograph shows (a) scope view of the laminar edge. The arrow shows the inferior edge of the lamina. (b) Scope view of the dural sleeve (white arrow) and nerve root (black arrow) (c) Scope view of the sequestrated disc (black arrow)

Once the endoscope was inserted the first step was the orientation of the image. A proper image orientation occurs when the underlying anatomy show the medial part on the top of the screen (12 o'clock) and the lateral one on the bottom (6 o'clock). One could accomplish this by placing a surgical instrument in a lateral position and then rotating the orientation ring on the camera/coupler until the instrument appears to be on the bottom of the video screen. The inferior edge of the lamina [Figure 2a] was identified after removing the soft tissues by coagulation and rongeur. The ligamentum flavum below the inferior edge of the lamina was identified and with the help of penfield the space was created between the flavum and the lamina. The overhang lamina was removed with the help of Kerrison rongeur till the edge of the flavum is reached. The flavectomy is done by punches after protecting it from the underneath dura. If required for this maneuver the flexible arm could be loosened to move the tubular retractor up and down. This was called “wanding” of the retractor. Once the flavectomy was done, the dural margin and nerve root were identified [Figure 2b]; the nerve root was then gently retracted. If there was large disc, tight root, sequestrated disc or lateral recess stenosis, the laminoforaminotomy could be widened for adequate root decompression. After retraction of the root, epidural dissection was carried out. The veins could be coagulated with bipolar coagulation. Once the disc space was reached, the sequestrated pieces could be removed or if annulotomy was required then it could be carried out with a micro-knife [Figure 2c]. Any loose pieces inside the disc space were removed with disc forceps. After discectomy, the final check of the root mobility was done. Entry port needs to be planned accordingly. Sometimes wanding of the scope was required to reach the site of sequestration or angulation required for reaching the central disc area.

Closure was done after a thorough wash and the dura was covered with a gelfoam. The scope was removed and the lumbodorsal fascia was sutured. Subcuticlar skin sutures were taken and dressing was applied.

All the patients except three were operated by an 18-mm tubular retractor. After enough experience with an 18-mm port, the last three patients were operated by a 16-mm tubular retractor. The patients were allowed to walk as soon as the patient was comfortable and surgical pain decreased. The patients were discharged between 24-48 h. Patients were encouraged walking till pain tolerance for 3 weeks. They were allowed all activities except bending forward, lifting weight and sitting for more than 30 min. Bending forward and lifting weight were restricted till 3 months postoperative. They were allowed to return to work after 3 weeks. The patients were followed up after 2, 6 and 12 weeks. The mean follow up was 12 months, (range 3 months-4 years). They were evaluated for symptoms of back pain, leg pain, and neurological deficit. Any new symptoms, complications of surgery, or the need for conversion to open surgery were also evaluated. The results were graded as excellent, good, fair, or poor depending on relief of back and leg pain, use of analgesics, and any complications. We have used modified Macnab criteria for grading the results. Excellent - no pain/restriction of activity and being able to do all activities; good - occasional pain with relief of presenting symptoms and returning to work with some modification; fair - some improved functional capacity but still handicapped or unemployed and poor results-having objective symptoms of root involvement or repeat surgery at the index level. The results were reviewed by the authors and not by an independent reviewer.

## RESULTS

**Figure 3:**
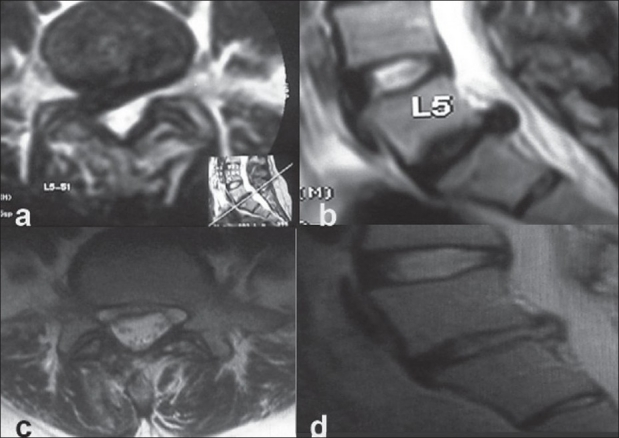
Preoperative T2WI axial (a) and saggital view (b) shows disc prolapse at L5-S1. Post operative T2WI axial (c) and saggital (d) shows adequacy of decompression

**Figure 4:**
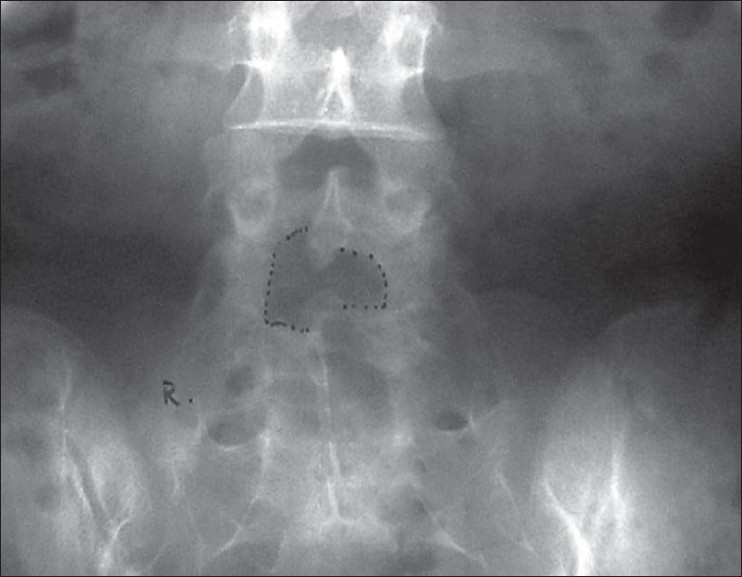
Postoperative X-ray lumbosacral spine anteroposterior view showing size of laminotomy

Surgery was successfully completed in all the patients. One patient with a nerve root injury required conversion to open surgery. The mean duration of surgery was 70 minutes (ranged 25 to 210 min). Peroperative complications were inadvertent removal of the facet joint (n=5), minor dural punctures (n=7) which did not require repair or open surgery conversion, and nerve root damage (patient=1) which required open surgery conversion. None of these patients had any clinical problem in the postoperative period. All the patients were discharged after 24-48 h of surgery. Up to 3 weeks, patients had some residual back or leg pain. The post operative MRI of first 11 patients showed complete decompression [Figure 3]. The post operative X-rays were also taken to see the size of laminotomy required [Figure 4].

We did have complications and difficulty in our initial cases. Four patients had postoperative discitis. Out of these four patients, three were treated conservatively and were relieved of their symptoms on the last follow-up and one patient had to be operated which required debridement and interbody fusion. One patient had nerve root damage during surgery, required an open surgery conversion but that patient had residual anaesthesia in L5 distribution in the last follow-up. Four patients had recurrence of disc at the same level. Recurrence occurred within 2 months of primary surgery. All the patients had an initial symptom-free period after surgery but developed recurrence of symptoms after that period. Repeat MRI showed recurrent/residual disc at the same level. Out of these, three patients had to be reoperated. One patient refused a second surgery but had fair results on the last follow-up. A total of 78 patients had an excellent outcome, 13 patients had a good outcome, 5 patients had a fair outcome and 4 had a poor outcome requiring repeat surgery. Overall, 91% of patients had excellent-to-good results. There were technical problems encountered during initial cases in the form of scope vision, image orientation, guide wire penetrating the dura, wrong level on either upper or even opposite side especially in obese patients and too lateral entry over the facet joints. All these problems were encountered in initial 25 cases.

## DISCUSSION

The overall results of standard discectomy range from 68% to 95% in different series.^12^–^16^ Though the results of standard discectomy are equally good, microdiscectomy introduced by Yasargil and Caspar (1977) is considered a gold standard. The results of microdiscectomy also range from 88% to 98.5%.^17^–^19^ Both the procedures are time-tested procedures giving a good surgical result in patients having disc prolapse. Katayama *et al*.^20^ compared the results of macrodiscectomy versus microdiscectomy. They concluded that there was no difference between the surgical outcome of both of them but microdiscectomy gave better lighting, magnification and therefore decreased the length of incision and tissue invasion. They also found that microdiscectomy allowed the patients to return early to work with lesser use of postoperative narcotic analgesics. It is but natural that if both the procedures have overall same outcome than the procedure with lesser tissue invasion, lesser length of incision, lesser use of postoperative analgesics with an early return to work is the procedure of choice.

MED introduced by Foley *et al*. combines standard lumbar microsurgical techniques with an endoscope, enabling surgeons to successfully address free-fragment disc pathologic factors and lateral recess stenosis. The endoscopic approach allows even smaller incisions and less tissue trauma, compared with standard open microdiscectomy. Because the MED procedure causes significantly less iatrogenic injury to the paraspinal musculature, it may potentially provide additional long-term benefits over more aggressive open procedures. The only thing which requires to be established is the long-term result comparable to standard microdiscectomy and the lesser tissue invasiveness than microdiscectomy.

Many reports are presented which prove the efficacy of MED with overall comparable results.^21^–^25^ Our study had an overall result of 91%. We compared our results with the series of Perez-Cruet *et al.*^21^ (n=150) where the average surgical time was 66 min, average blood loss was 22 ml, average hospital stay was 7.7 h, complication rate was 5%, reoperation rate was 4%, and average return to work was 17 days with an overall result of 94%. We had 24-48 hrs of hospital stay compared to 7.7 h of their study. Other factors like surgical time (66 vs. 70 min), complication rate (5% both series), reoperation rate (4% vs. 3%), return to work (17 vs. 21 days), and overall results (94% vs. 91%) are comparable in both series. Similar results are reported by Ranjan *et al*.^24^ in their series of 107 cases. Their average surgical time was 120 min, hospital stay was of 24–48 h, complication rate was 6.5% with open surgery conversion in one patient and recurrence in two patients. Our series had one open surgery conversion and recurrent disc in four patients (4%). From these data, it can be concluded that MED is safe and effective. As yet, there is no good prospective randomized study to compare the results of MED, microdiscectomy, and standard discectomy. Though there is one nonrandomized study by Schizas^26^ which compared the results of MED with standard microsurgical discectomy and concluded that MED is at least as effective as microsurgical discectomy for the treatment of uncontained or large contained disc herniations.

Microendoscopic discectomy (MED) has claimed even lesser tissue invasion than microdiscectomy with even smaller skin incision, lesser use of analgesics, and early return to work. Least tissue invasion is established by many reports comparing the postoperative MRI signal of paraspinal muscles,^27^ intraoperative EMG findings establishing less invasion to nerve roots,^28^ and by measuring serum levels of biochemical parameters reflective of a postoperative inflammatory reaction and damage to the paravertebral muscles.^29^ Our personal opinion is similar as all patients had only a 18-mm skin incision and postoperative MRI done in initial cases showed very less signal changes in the paraspinal muscles though these were not the parameters studied in our series.

Minimally invasive microendoscopic decompression technique has been used not only for paracentral discectomies but also for lateral disc herniations,^30^ recurrent disc her niations,^31^ decompressions of lumbar canal stenosis^32^ and transforaminal interbody fusion.^33^

In our series, the complication rate is 5% and the recurrence rate is 4% which also match with the results of macro- and microdiscectomy. The complications which we had are due to initial learning curve. MED has a definite learning curve because of two-dimensional visions, orientation with scope, handling of the scope, less space available for dissection, and managing epidural bleeding.^34^,^35^

Though from our initial experience, it seems MED is a technique which gives early rehabilitation and less bleeding.The limitation of this study has been lack of comparable control to compare and quantify that in MED there is less bleeding and early rehabilitation compared to standard or microdiscectomy. A well-designed double-blind prospective randomized control trial needs to be done comparing MED and microdiscectomy and standard discectomy to prove these facts.

## CONCLUSION

Microendoscopic discectomy is a minimally invasive procedure for discectomy with early encouraging results. It has a learning curve initially but once expertise is acquired over the technique, the results of this procedure are acceptable and are safe and effective.
